# Electrophysiological assessment of nutritional optic neuropathy: a case report

**DOI:** 10.1007/s10633-022-09914-7

**Published:** 2023-01-19

**Authors:** Emily K. O’Neill, Kshitij Mankad, Richard Bowman, Dorothy A. Thompson

**Affiliations:** 1 Clinical and Academic Department of Ophthalmology, Visual Electrophysiology Unit, Great Ormond Street Hospital for Children, NHS Trust, 40-41 Queen Square, London, WC1N 3AJ UK; 2 Department of Radiology, Great Ormond Street Hospital for Children, NHS Foundation Trust, London, UK; 3 UCL Great Ormond Street Institute of Child Health, University College London, 30 Guildford Street, London, UK

**Keywords:** Visual evoked potential, Electroretinogram, Nutritional optic neuropathy, Vitamin A deficiency, Optic canal hyperostosis

## Abstract

**Purpose:**

To report an unexpectedly asymmetric, progressive nutritional optic neuropathy associated with vitamin A deficient optic canal hyperostosis in a 15-year-old female with a long history of a restricted diet.

**Methods:**

We performed comprehensive ophthalmic assessments in a fifteen-year-old female with a long history of restricted eating who presented with suspected nutritional optic neuropathy, predominantly affecting the right eye vision.

**Results:**

A review of computerised tomography and magnetic resonance imaging revealed bilateral optic canal hyperostosis likely associated with vitamin A deficiency. Electrodiagnostic tests and optical coherence tomography provided structure–function evidence of bilateral retinal ganglion cell dysfunction and notably revealed severe loss of temporal fibres in the left eye which showed cecocentral scotoma but normal visual acuity. Although selective damage of the papillomacular bundle has been well-documented in nutritional and toxic optic neuropathies, compressive optic canal hyperostosis secondary to nutritional deficiency has been rarely reported.

**Conclusions:**

Nutritional deficiencies are increasing in high-income countries and may be linked to the rise of gastrointestinal disorders, strict vegan and vegetarian diets and avoidant restrictive food intake disorder (ARFID) associated with conditions such as depression and autism spectrum syndrome (ASD). Our findings highlight the value of electrodiagnostic testing alongside imaging in complex nutritional optic neuropathies to help monitor, guide treatment and preserve remaining sight in a child.

## Introduction

A fifteen-year-old girl underwent investigations for a progressive, painless uniocular vision loss that reduced her right eye (RE) to light perception (LP) over a 2-year-period. A RE relative afferent pupil defect and distant exotropia 40∆ were noted. Left eye (LE) visual acuity (VA) was preserved at LogMAR 0.00, but colour vision was poor (Ishihara test plate only). Fundus exam revealed bilaterally normal maculae and peripheral retina, but bilateral optic nerve pallor (3 + right and 1 + left on the Frisén Scale). A cecocentral scotoma was described in her LE, and visual field assessment was not possible with her RE. Bitot spots were identified.

The patient described a long history of a diet restricted to potatoes and crisps. She had iron deficiency anaemia and was deficient in vitamins B12 and D, and copper with limited adherence to supplementation. Vitamin A levels were significantly low (0.11 µmol/L, reference range 0.90—2.50 µmol/L). The patient had no allergies, gastrointestinal issues, or family history of ophthalmic or medical pathology.

### Electrodiagnostic and imaging assessment

The patient underwent ISCEV pattern ERG (PERG) [1], pattern VEP (PVEP), flash VEP [2] and flash ERG [3, 4] recordings alongside ophthalmic imaging within the GOSH Ophthalmology department.

## Methods

PVEPs were recorded from a three-channel trans-occipital array (O1, Oz, O2) referenced to Fz (mid-frontal). PERGs were simultaneously recorded with PVEPs using DTL fibre electrodes referenced to outer canthus. Lower-lid skin ERGs were used for flash ERG recording. Impedances remained equal and below 5KΩms, and fixation was monitored throughout using closed circuit TV. Repeated trials were attained to ascertain repeatability.

Pattern reversal and flash ERGs and VEPs were recorded using Diagnosys Espion hardware and E6 software version number V6.64.9 with amplifier input gain 8. A filter bandpass of 0.3–100 Hz was used with an acquisition time window 15 ms pre-stimulus and 285 ms post-stimulus. The sample frequency was 1 kHz, with a pattern reversal rate of 3.15 Hz for PERG and PVEP recording. Pattern reversal checkerboards (check widths 6.25’-400’) including the ISCEV [2] large and small check widths were presented 3 rev/second on a plasma display panel of mean luminance 82 cd/m^2^ with 30*°* field size a viewing distance of 1 m. A series of different flash strengths were presented under scotopic and photopic conditions, incorporating blue and red flashes, using a Grass PS33 handheld strobe.

Flashes were presented 3 Hz, 3/s or (1 flash per 330 ms) except for the maximum flash which had a 20 s ISI and the 30 Hz flicker stimulus. Corneal DTL electrodes were referenced to gel electrodes placed on the outer canthus for PERG recording. VEPs were recorded from Ag/AgCl cup electrodes Skin preparation with Nuprep, conductive Elefix paste and secured with Coban 3 M headband.

## Results

**Fig. 1:**
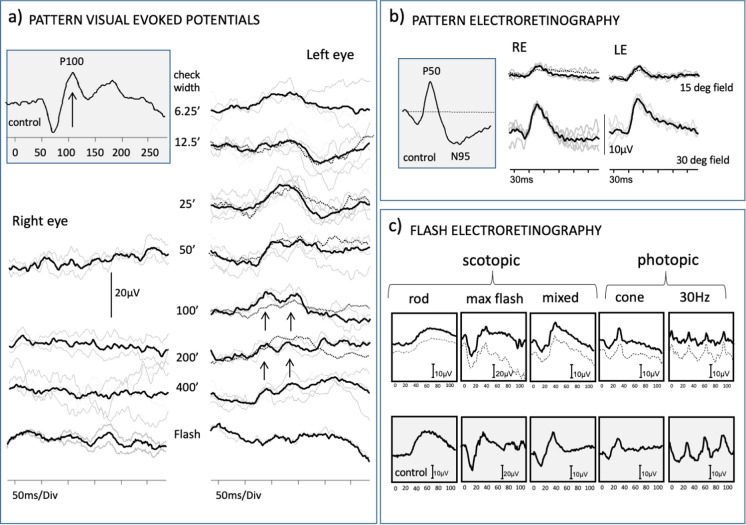
Electrophysiological findings in the patient with laboratory control traces for reference (grey boxes). Individual trials are shown in grey and average waveforms in black. **a** Monocular pattern reversal visual evoked potentials (prVEPs) from the mid occiput captured in March 2020 (average shown as a solid black line), overlaid with responses acquired in 2021 when vitamin A levels resolved (dotted line). Pattern reversal VEPs presented in descending order for spatial frequency (minutes of arc) including ISCEV standard small and large check widths from each eye in a 30° field, showing inter-ocular difference. Right eye prVEPs are indistinguishable from background noise to 50’, 200’ and 400’ check widths and LE prVEPs are evident to a range of check widths but appear atypically bifid to larger check widths (black arrows). A bifid PrVEP waveform is a feature of central scotoma or reduced sensitivity of the central field, such as associated with maculopathy of optic atrophy [5, 6]. **b** Monocular pattern electroretinography (pERG) presented to 50’ check widths to each eye in 30° and 15° fields. Individual trials are shown in grey and average waveforms in black. PERG P50 components from each eye were within laboratory reference range, but each eye showed markedly reduced N95 components and thus abnormally small N95:P50 ratios. PERG data from 22 months after initial presentation, when vitamin A levels were resolved, are overlaid for the small field. **c** Photopic flash ERGs obtained through GOSH protocol using a Grass PS33 handheld strobe at setting 4 captured in October 2019 (dotted line) and March 2020 (solid line) with individual trials shown in grey. Scotopic ERGs are within reference range; however, LE photopic cone b-wave amplitude and b:a ratio are reduced relative to the RE

Flash ERGs were within normal reference limits (Fig. 1c). PERG N95 components showed a marked bilateral selective reduction of N95 amplitudes from 30- and 15-degree fields, (Fig. 1b). PRVEPs produced by the LE to a range of check widths had bifid waveforms (arrowed Fig. 1a). PRVEPs from the RE were indistinguishable from the background noise. Flash VEPs from each eye were degraded and attenuated.

**Fig. 2:**
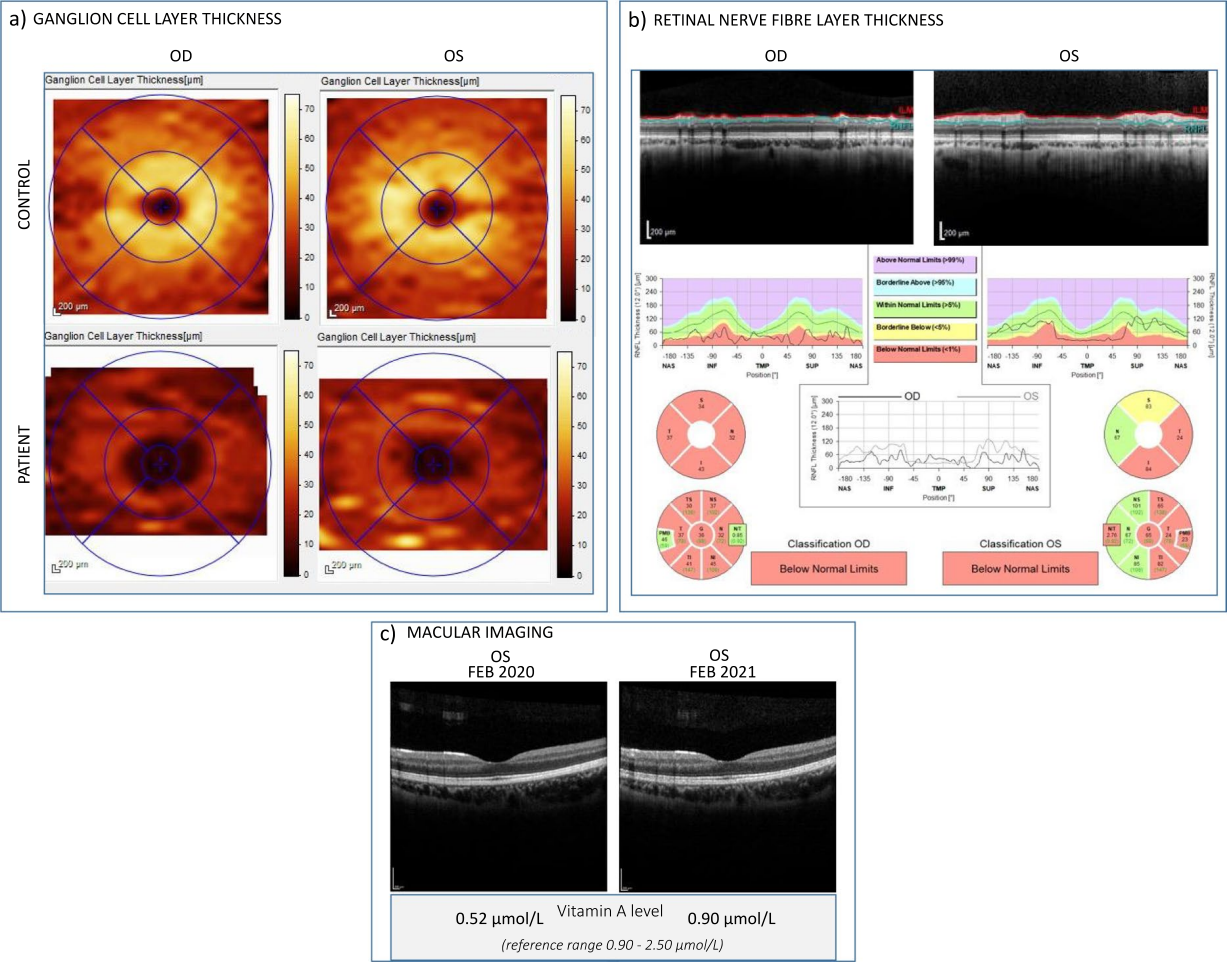
Optical coherence tomography (OCT) ganglion cell layer (GCL) **a** and retinal nerve fibre layer (RNFL) **b** thickness reports of the patient taken in October 2019 and macular images of the left eye taken in February 2020 and 2021 **c**. Thickness reports show bilateral loss of both layers compared to a control patient. GCL loss appears equally severe in both eyes; however, RNFL loss is widespread and severe in the right eye but confined to the temporal hemi-sector within the left eye. OCT RNFL findings were consistent and stable with consecutive axonal reports conducted in 2020 and 2021. These findings illustrate the benefit of combining GCL and RNFL analysis. Macular scans of the left eye **c** do not demonstrate clear structural abnormalities and are comparable when vitamin A levels were reported to be reduced and resolved. Vitamin A levels shown are taken from the latest laboratory tests available for the patient at the time of image capture; January 2020 and December 2020, respectively

Optical coherence tomography (OCT) (Spectralis, Heidelberg) analysis of the retinal nerve fibre layer (RNFL) showed marked thinning of all sectors of the RE RNFL, whilst thinning of the RNFL in the LE was limited to the temporal hemi-sector (Fig. 2b).

The asymmetric loss of vision was unusual for nutritional optic neuropathy, and other diagnoses were considered. Abetalipoproteinemia was dismissed through the lack of characteristic retinal pigmentation, ataxia, and dysarthria. Neurological assessment did not identify any abnormalities, and investigations for syphilis, borrelia and tuberculosis were negative. No antibodies for Aquaporin 4 or myelin oligodendrocyte glycoprotein were detected. Genetic analysis for Leber’s hereditary optic atrophy and autosomal dominant optic atrophy (OPA1) showed no pathological variants.

**Fig. 3:**
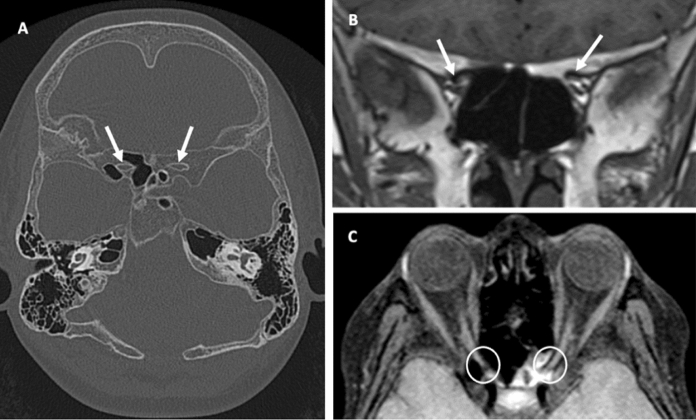
Selected neuroimaging views from the patient. Axial head computed tomography (CT) (**A**) demonstrates generalised expansion of the diploic spaces causing secondary narrowing of the optic canals which is fairly symmetrical (arrows). Coronal T1 weighted magnetic resonance imaging (MRI) (**B**) and axial fat-suppressed T1 weighted MRI (**C**) show flattening and attenuation of the intracanalicular aspects of the optic nerves bilaterally (arrows)

Historic MRI and CT images were reviewed. On the CT, diffuse expansion of diploic spaces was noted to cause fairly symmetrical secondary narrowing of both proximal optic canals (Fig. 3a). On the two MRIs previously taken, intracanalicular optic nerves in the optic canals appeared slightly flattened and compressed by the optic canal narrowing. These features suggested compressive elements were contributing to the bilateral asymmetric optic neuropathy. Bilateral optic narrowing secondary to hyperostosis was reported, with vitamin A deficiency proposed as a possible mechanism.

## Discussion

The asymmetric vision loss was atypical for nutritional optic neuropathy. Classically, patients experience bilateral progressive painless vision loss with central or cecocentral scotomas, reflecting selective damage to the papillomacular bundle [7]. Patients typically show early dyschromatopsia, impaired contrast sensitivity and temporal optic disc atrophy [8]. Our patient was deficient in many of the nutrients essential for neurological function, including B1, B9, B12 and copper, which contribute to mitochondrial health [9, 10].

Compression caused by optic canal hyperostosis, likely associated with secondary vitamin A deficiency, may have contributed to the uncommon asymmetrical presentation. Although the underlying mechanisms are unknown, bilateral bony compression can have unilateral effects of functional consequence, for instance, in osteopetrosis [11]. Compression along the optic canal may differentially disrupt the nerves, and there may be a spatiotemporal relationship between compression and atrophy which may have aided the unique preservation of papillomacular nerve fibre bundle for the left eye. Although it is well-understood that vitamin A deficiency in immature mammals can impact bone structure and can lead to skull hyperostosis, optic nerve compression and blindness, it has rarely been reported in humans [12–14].

We compared our patient’s profoundly low vitamin A levels (0.11–0.32 µmol/L, reference range 0.90—2.50 µmol/L) with published cases (Table 1) to ascertain the minimum threshold for rod ERG alterations and other clinical signs such as Bitot's spots. Although Bitot’s spots were observed, the patient had not reported any nyctalopia and rod system ERGs were normal, while vitamin A levels were deficient (0.32 µmol/L reported the day before testing). Rod ERGs are an important tool to detect VAD; however, few cases of rod ERG depression have been published, and there is variability in vitamin A levels reported in the literature before the rod ERG is affected (Table 1). Rod ERGs have been reported to be subnormal when vitamin A levels are 0.1–0.19 µmol/L, with rod ERGs described as normal at > 0.2 µmol/L (Table 1), levels which remain below our institute’s vitamin A reference interval. As our patient periodically received supplementary vitamin A injections, it is possible that it is chronic deprivation in contrast to pulsed levels which deplete the retina and manifest within ERG testing. Vitamin A deficiency has been shown to inhibit the mobilisation of iron stores in tissues [15] and thus may contribute to the patient’s long history of iron deficiency anaemia. Interestingly, there has been a report of optic canal stenosis associated with congenital haemolytic anaemia [16]. Although vitamin B12 deficiency rarely manifests visual symptoms, it can cause bilateral optic neuropathy and optic atrophy, which is typically symmetrical [17]. In the context of possible contributions to the patient presentation, we have detailed the patient’s B12 levels throughout the observation period (Table 2).

**Table 1:** An overview of published vitamin A levels in nutritional retinopathy literature alongside those reported in this study

Authors, Year	Age(s)	Vitamin A level, with age-matched reference values	Rod ERG	Comments
Russell, 2000 [25]	N/A	> 1.4 umol/L was shown to predict normal dark adaptation 95% of the time	N/A	'Other causes of abnormal dark adaptation include zinc and protein deficiencies
Ramsey, 2001 [26]	7	0.35 umol/L Reference range 0.4–1.1 umol/L	Normal for each eye	Bitot spots reported
McBain, 2005 [27]	59, 55, 9	0.1 |imol/L Reference range 1.5–2.5umol/L 0.19umol/L Reference range 1.05–2.80umol/L 0.1 umol/L Reference range 0.9–1.7 umol/L	Bilaterally undetectable (scotopic rod) ERGs at brighter intensities resembled those derived solely from the cone system	Cone ERGs in 2/3 cases were increased in latency and borderline/subnormal amplitude
Chiu and Watson, 2015 [28]	12	< 0.4 //mol/L Reference range 0.9–2.5 //mol/L0.2umol/L Reference range 0.84–3.6umol/L	Not reported	'Electrophysiology confirmed bilateral optic nerve dysfunction
Zayed, 2015 [12]	17	< 0.1umol/L Reference range 0.90–1.7umol/L	Normal for each eye	'Left eye pattern ERG was attenuated with an absent cortical pattern visual evoked response, consistent with a left optic neuropathy
Klnlln, 2019 [13]	10	Undetectable	N/A	Bitot spots reported. MRI showed bilateral papilledema and possible narrowing of the optic canals
Raouf, 2021 [14]	15	N/A	Requested, never obtained	Worsening bilateral vision, worse at night
This study	16	Normal for each eye	Normal for each eye	Bitot spots reported Patient had received two vitamin A injections (50,000 units) between vitamin A test and ERG

**Table 2:** The patient’s serum vitamin B12 and vitamin A levels reported over the observation period. Note that the patient received vitamin B12 and A supplementation (L: Lower than reference range, H: Higher than reference range)

	2019			2020					2021	
	July	August	October	January	April	June	October	December	April	September
Serum Vitamin B12 Reference range 182–820 ng/L	> 1,000(H)	** > 1**,000 (H)	** > 1,**000(H)	** > 1**,000 (H)	843 (H)	780	892 (H)	655	695	684
Vitamin A Reference range 0.9–2.5 fjmol/L	0.09 (L)	0**.11** (L)	0.32 (L)	0.52 **(L)**	0.63 (L)	0.94	0.75 **(L)**	0.90	1.12	0.85 (L)

It is well established that patients with VAD may exhibit structural retinal changes detected through OCT, which often normalise following vitamin A supplementation. These include reports of outer retinal abnormalities, including thinning of the outer nuclear layer, disruption of the photoreceptor IS/OS junction [18] and foveal hyperreflectivity anterior to the retinal pigment epithelium-Bruch’s complex recently termed the ‘double carrot’ sign [19]. These findings have mostly been reported in older male patients following bariatric surgery, often with additional complications, and are not clearly evident within our patient (Fig. 2c).

Early detection of nutritional causes of sight loss is essential as supplementation can restore visual function in initial stages of disease, but a holistic approach to treatment is recommended due to the well-documented role of social and behavioural factors [20]

Rising cases of nutritional optic neuropathy and retinopathy in high-income countries are attributed to malabsorption syndromes caused by inflammatory bowel diseases and gastric bariatric surgery, popularity of strict vegan and vegetarian diets, high rates of alcoholism, and avoidant restrictive food intake disorder (ARFID) associated with conditions such as depression and autism spectrum syndrome (ASD) [21]. Indeed, eye disorders secondary to vitamin A deficiency in ASD are increasing and screening of vulnerable paediatric populations may prevent treatable sight loss [22, 23]. It is important to consider that single vitamin deficiencies rarely occur in isolation [24], and often ASD patients will be at risk of many coexisting micronutrient deficiencies.

The specific aetiology of disease in this patient is still uncertain and complicated through multiple deficiencies and compressive elements. Visual loss in this case is likely a consequence of multiple interactions of multi-nutrient deficiencies, including vitamin B12, which may underlie the optic neuropathy seen in this patient. The asymmetric presentation of our patient is rare and proposes complexities which may not be fully realised through our investigations. Optic nerve and visual pathway function of both eyes were severely affected at presentation despite the reported normal LE VA. Poor adherence to nutritional supplementation makes prognosis difficult, but eating behaviours seemed to change positively after the patient was given psychological support. The patient disclosed that her selective eating behaviours are associated with contamination fears, obsessive behaviours including excessive hand washing and past trauma.

This unusual presentation of severe unilateral vision loss associated with optic canal hyperostosis secondary to nutritional deficiencies highlights the value of multimodal functional and structural measures in complex nutritional optic neuropathies. Potential optic nerve compression should be considered in the aetiology of nutritional optic neuropathy.

## Declarations

### Statement on the welfare of animals

This article does not contain any studies with animals.
